# The Kynurenine/Tryptophan Ratio Is a Sensitive Biomarker for the Diagnosis of Pediatric Tuberculosis Among Indian Children

**DOI:** 10.3389/fimmu.2021.774043

**Published:** 2022-01-12

**Authors:** Jeffrey A. Tornheim, Mandar Paradkar, Henry Zhao, Vandana Kulkarni, Neeta Pradhan, Aarti Kinikar, Anju Kagal, Nikhil Gupte, Vidya Mave, Amita Gupta, Petros C. Karakousis

**Affiliations:** ^1^ Center for Tuberculosis Research, Department of Medicine, Johns Hopkins University School of Medicine, Baltimore, MD, United States; ^2^ Center for Clinical Global Health Education, Department of Medicine, Johns Hopkins University School of Medicine, Baltimore, MD, United States; ^3^ Byramjee Jeejeebhoy Government Medical College-Johns Hopkins University Clinical Research Site, Pune, India; ^4^ Johns Hopkins University, Baltimore, MD, United States; ^5^ Byramjee Jeejeebhoy Government Medical College, Pune, India; ^6^ Department of International Health, Johns Hopkins Bloomberg School of Public Health, Baltimore, MD, United States

**Keywords:** biomarker, transcriptomics, metabolomics (OMICS), diagnostics, pediatric tuberculosis

## Abstract

**Objectives:**

Pediatric tuberculosis (TB) remains difficult to diagnose. The plasma kynurenine to tryptophan ratio (K/T ratio) is a potential biomarker for TB diagnosis and treatment response but has not been assessed in children.

**Methods:**

We performed a targeted diagnostic accuracy analysis of four biomarkers: kynurenine abundance, tryptophan abundance, the K/T ratio, and IDO-1 gene expression. Data were obtained from transcriptome and metabolome profiling of children with confirmed tuberculosis and age- and sex-matched uninfected household contacts of pulmonary tuberculosis patients. Each biomarker was assessed as a baseline diagnostic and in response to successful TB treatment.

**Results:**

Despite non-significant between-group differences in unbiased analysis, the K/T ratio achieved an area under the receiver operator characteristic curve (AUC) of 0.667 and 81.5% sensitivity for TB diagnosis. Kynurenine, tryptophan, and IDO-1 demonstrated diagnostic AUCs of 0.667, 0.602, and 0.463, respectively. None of these biomarkers demonstrated high AUCs for treatment response. The AUC of the K/T ratio was lower than biomarkers identified in unbiased analysis, but improved sensitivity over existing commercial assays for pediatric TB diagnosis.

**Conclusions:**

Plasma kynurenine and the K/T ratio may be useful biomarkers for pediatric TB. Ongoing studies in geographically diverse populations will determine optimal use of these biomarkers worldwide.

## Introduction

Tuberculosis (TB) is a leading global cause of morbidity and mortality, and is likely to re-emerge as a primary cause of death from infection following the current global pandemic (1). Diagnosis in young children, however, remains a significant challenge due to their inability to produce adequate sputum samples, the frequency of extrapulmonary disease, and the overall paucibacillary nature of pediatric infections (2). In that context, many studies have sought to identify host-derived markers of infection in children that do not rely on direct detection in clinical samples of *Mycobacterium tuberculosis (*Mtb), the bacterium that causes TB (3–5). A recent series of studies highlights the increased kynurenine concentration and lower tryptophan concentration in the blood of patients with pulmonary TB (6, 7). The resulting ratio of kynurenine to tryptophan (K/T) has therefore been proposed as a potential biomarker for the diagnosis of TB that was also associated with treatment outcomes. The decline in tryptophan is associated with the induction of immunoregulatory enzyme indoleamine 2,3-dioxygenase (IDO-1), which breaks down tryptophan to kynurenine metabolites and suppresses the immune response, particularly through shifts in macrophage metabolism and induction of T-cell anergy and apoptosis (8, 9). Metabolic changes in this pathway have also been identified among those with latent TB infection, as well as in multidrug-resistant TB, cavitary disease, and extrapulmonary TB, with low tryptophan in the cerebrospinal fluid of people with tuberculous meningitis (7, 10). Moreover, changes in tryptophan catabolism returned to normal during TB treatment, suggesting a role as a marker of TB disease activity and treatment response. The K/T ratio and IDO-1 activity have been associated with diagnosis of TB and response to treatment in other special populations of interest, such as people with HIV (11), but not yet in children. In order to assess the roles of kynurenine, tryptophan, the K/T ratio, and IDO-1 gene expression as diagnostic biomarkers and indicators of successful treatment response, we performed a secondary analysis of data from two previous studies of pediatric transcriptomic and metabolomic profiling (3, 4).

## Materials and Methods

The data were initially collected as part of a nested laboratory substudy from a 5-year prospective observational cohort of adults and children with tuberculosis (cases) and the household contacts of participants with pulmonary tuberculosis (controls) described in previous publications (12). Study participants were enrolled at the Byramjee Jeejeebhoy Government Medical College (“BJGMC”), a tertiary teaching hospital in Pune, India in collaboration with researchers from Johns Hopkins University. Samples were collected from study participants at the time of enrollment, as well as longitudinally throughout treatment for cases and for 1 year after enrolment for controls. Study participants under 15 years of age from Pune, India with confirmed tuberculosis had PAXgene tubes selected from the study biorepository for unbiased transcriptomic analysis (3) and plasma samples selected for integrated unbiased metabolomic analysis (4). Participants had tuberculosis confirmed by a combination Xpert MTB/RIF (6 participants), culture (6 participants), or the presence of granulomas on histopathology of extrapulmonary specimens (7 participants, not mutually exclusive). All cases were successfully treated to cure with a combination of isoniazid, rifampin, pyrazinamide, and ethambutol, and each case was age and sex-matched with 2 controls for these analyses. All cases and controls were HIV negative and all controls were ruled out for active tuberculosis at the time of enrolment by symptom screen and chest X-ray, and rule out for latent tuberculosis infection (LTBI) by tuberculin skin test (TST) and interferon gamma release assay (IGRA) at the time of enrolment. Whole blood samples were collected from cases for transcriptional profiling at baseline, 1 month, and 6 months of treatment, and from controls at enrolment, month 4-6, and month 12 after enrolment. Plasma samples were collected on the same schedule for metabolomic profiling. TST and IGRA testing were repeated at each visit to identify new latent or active tuberculosis over the first 12 months after enrolment. No controls developed active TB during that period.

Transcriptional profiling was performed after RNA isolation from PAXgene tubes and sequenced by Illumina HiSeq 2500, aligned to the human genome (GRCh38.10) using the STAR aligner and annotated using GENCODE (13). Differential expression analysis was conducted in R using DESeq2. This analysis identified a 71-gene diagnostic signature and a 25-gene treatment response signature for pediatric tuberculosis. Metabolomic profiling was performed with the automated MicroLab STAR system using Waters ACQUITY ultra-performance liquid chromatography (UPLC), a Thermo Scientific Q-Exactive high resolution/accurate mass spectrometer and Orbitrap mass analyzer operated at 35,000 mass resolution with a scan range of 70-1000m/z (14, 15). Peaks were identified using a standardized commercially available library of known compounds (16). Differentially expressed metabolites were identified between groups and a random forest decision tree identified the simplest combinations of metabolites that differentiated groups with the greatest accuracy. Integrated multi-omics analysis identified the relative contributions of metabolomic and transcriptomic data to the optimal features that diagnosed tuberculosis and identified treatment response in children. To control for multiple comparisons, differential expression and abundance was defined as ≥2-fold difference between groups with a Benjamini-Hochberg false discovery rate of <0.05 applied to correct for multiple comparisons. Data from those studies are available from NCBI (accession code PRJNA588242) and in the supplementary files of our prior publications (4).

In the present analysis, we combine the two published datasets to determine the extent to which the tryptophan, kynurenine, the K/T ratio, and IDO-1 were able to discriminate groups of children with and without TB (i.e., as a diagnostic biomarker), and children with TB over time from the start of treatment, after 1 month of treatment, and at the end of 6 months of successful treatment (i.e., as a biomarker of treatment response). Relative abundance of metabolites and gene expression levels were correlated between participants and summarized by R^2^ levels. Relative abundance between study groups and time points was assessed by Wilcoxon tests for paired samples and the Kruskal-Wallis test for multiple groups, with differences of p<0.05 considered significant. Receiver operator characteristic (ROC) curves were calculated for each potential biomarker using the pROC package in R with optimal thresholds determined by Youden’s index and overall accuracy presented by area under the ROC curve (AUC) [25].

## Results

### Participants

Participants with TB had a median age of 8.5 years (interquartile range, IQR=6.8-12), half were male, and half had only pulmonary tuberculosis only. All cases and controls were HIV seronegative. No controls had active TB or latent TB infection at the time of enrollment, and none developed active TB in the subsequent year, but 13 (40.6%) developed positive TST or IGRA during the study.

### Correlation Between Biomarkers and Unbiased Analysis

**Figure 1:**
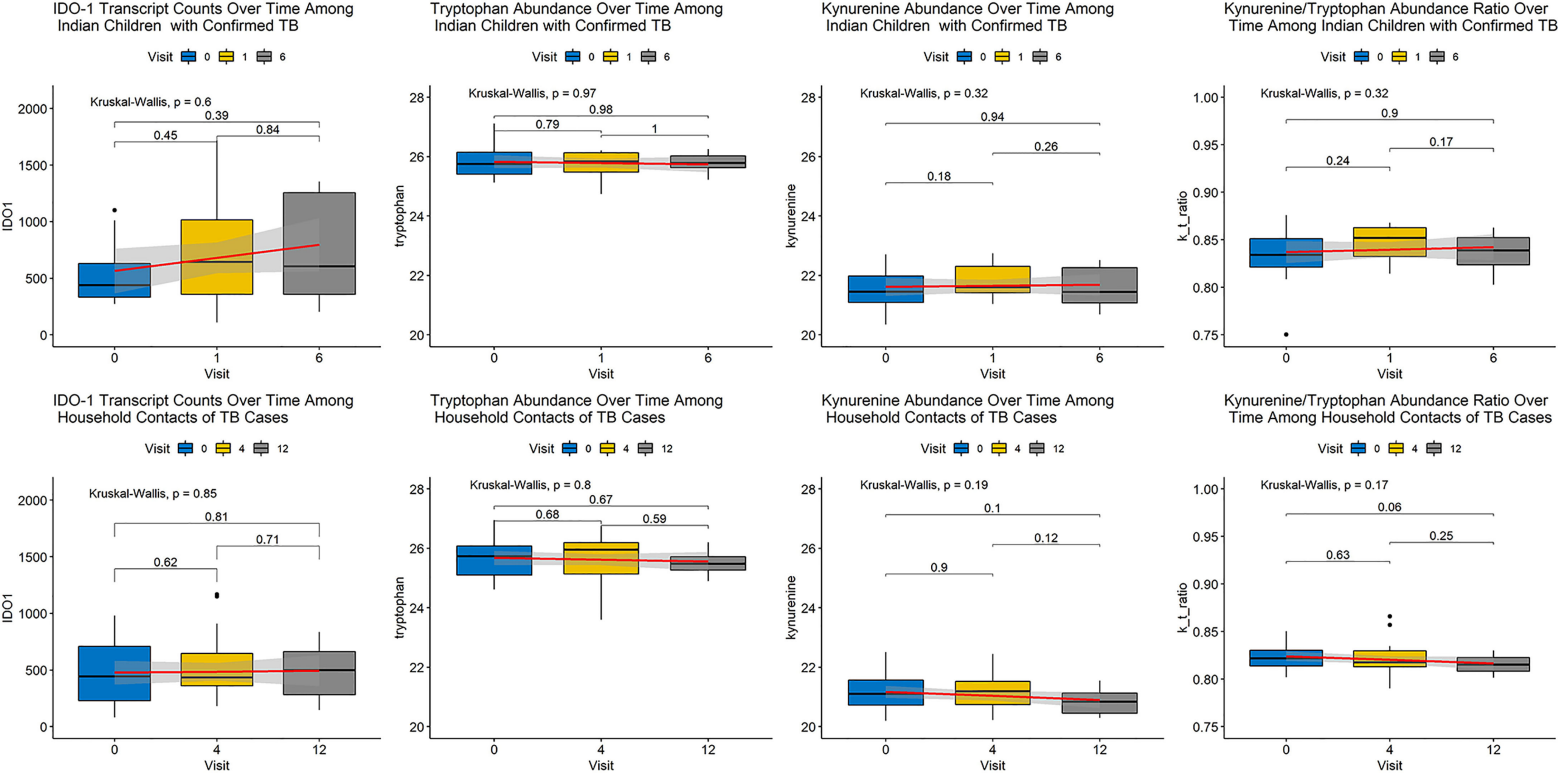
Changes in Metabolite Abundance and Transcript Expression Over Time Among Cases and Controls. Abundance of each biomarker of interest is expressed as a boxplot with a smoothed regression line in red indicating change over time at each visit, indicated by month of visit. Shading around each red line indicates 95% confidence around the trend. The top row of panels contains plots for cases and the bottom row of panels indicates plots for controls. Each column represents a distinct biomarker with IDO-1 transcript expression in the left column, followed by tryptophan abundance, kynurenine abundance, and the kynurenine/tryptophan (K/T) ratio moving from left to right. None of these biomarkers demonstrated a significant change over duration of follow-up among cases or controls.

Tryptophan and kynurenine abundance were poorly correlated with contemporaneous IDO-1 transcript abundance across all study time points (R^2^<0.001 and R^2^ = 0.014, respectively), with similar poor correlation between IDO-1 and the K/T ratio (R^2^ = 0.023) and better correlation between tryptophan and kynurenine levels (R^2^ = 0.347). Kynurenine demonstrated a small but significant decrease among cases between the start and end of treatment (-0.029-fold, p=0.037), although this effect was not apparent until the completion of treatment (**Table 1** and **Figure 1**). Neither kynurenine or tryptophan abundance, nor IDO-1 expression was significantly different between any of the other groups compared for either diagnosis, treatment response, or incident tuberculosis infection among controls during the study period.

**Table 1:** Differential abundance and expression of kynurenine, tryptophan, and IDO-1 between study groups.

	Kynurenine Abundance		Tryptophan Abundance			IDO-1 Expression	
Groups Compared	Fold Change	Log_10_ p-value	Fold Change		Log_10_ p-value	Fold Change	Adjusted p-value
Cases vs. Controls	0.345	1.046	0.090		0.212	0.320	0.668
Cases Starting Treatment vs. Treatment Month 1	-0.263	0.600	0.120		0.283	0.273	0.585
Cases at Treatment Month 1 vs. End of Treatment	0.234	0.415	-0.044		0.101	-0.198	0.779
Cases Starting vs. Finishing Treatment	-0.029	0.037	0.077		0.181	-0.090	0.803^1^
Cases at the End of Treatment vs. New LTBI	0.473	1.097	0.094		0.177	2.362	0.769
Controls with New LTBI vs. Controls without New LTBI	-0.171	0.432	-0.138		0.250	0.902	0.531

^1^Adjusted p-value could not be calculated, unadjusted p-value presented.

### Biased Assessment for Diagnosis and Treatment Response

Next, we assessed each biomarker individually by ROC analysis and without controlling for multiple comparisons (as was performed in the unbiased analysis above). None of the biomarkers significantly differentiated cases from controls, though kynurenine abundance was higher among cases than controls (p=0.051). Despite non-significant differences between groups in unbiased analysis, kynurenine and the K/T ratio achieved good discrimination of cases from controls (AUC 0.667 and AUC 0.676 for kynurenine and the K/T ratio, respectively). These biomarkers performed less well as measures of treatment response, with AUCs of 0.494-0.606 (**Table 2**). Subgroup analysis did not find that discrimination ability improved when comparisons were limited to only controls without incident TB during follow-up. None of these biomarkers demonstrated consistent changes in abundance or expression over time by study group that would suggest use as an indicator of clinical improvement during treatment or as an early marker of incident latent infection in children (**Figure 1**).

**Table 2:** Test characteristics of kynurenine and tryptophan abundance, the K/T ratio, and IDO-1 expression for the diagnosis of pediatric tuberculosis and treatment response.

	Cases vs. Controls				Response to Treatment			
Groups Compared	AUC	Best Threshold	Sensitivity	Specificity	AUC	Best Threshold	Sensitivity	Specificity
Kynurenine Abundance	0.667	20.955	0.444	0.875	0.513	21.099	0.400	0.750
Tryptophan Abundance	0.602	25.329	0.407	0.938	0.494	25.550	0.800	0.375
K/T Ratio	0.676	0.833	0.815	0.563	0.519	0.842	0.500	0.688
IDO-1 Expression	0.463	648.5	0.333	0.750	0.606	1167	0.400	1.000

## Discussion

As the world recovers from the COVID-19 pandemic, it is likely that we will see a rise in reported TB cases in the coming years. Improved tools for the diagnosis of pediatric TB and non-sputum based markers of treatment response are needed to reduce the morbidity and mortality of these new cases. When new host biomarkers of tuberculosis are identified, it is imperative that these be assessed in special populations, including children, to confirm if and how they can be best employed. In this secondary analysis of metabolomic and transcriptomic data, we evaluated the performance of the following four biomarkers for the diagnosis of pediatric TB and assessment of response to successful treatment: kynurenine, tryptophan, the K/T ratio, and IDO-1 gene expression. We found that plasma kynurenine levels declined over the course of TB treatment, and that both plasma kynurenine and the plasma K/T ratio achieved AUCs >0.66, with the K/T ratio achieving a sensitivity of 82% and a specificity of 56% for diagnosis of pediatric TB in this population. The other biomarkers assessed did not perform as well for either diagnosis or response to treatment. This suggests that the K/T ratio has acceptable accuracy for use as a diagnostic tool in children with TB, meeting the WHO target product profile by achieving better sensitivity than existing commercial diagnostic tools for pediatric TB (17).

IDO-1 activity is a compelling target biomarker for TB diagnostics due to multiple studies demonstrating changes in this pathway in a variety of types of TB, as well as the availability of an inhibitor, 1-methyl-tryptophan, that has been associated with clinical improvement and increased *Mtb* killing (18). The absence of correlation between contemporary IDO-1 transcript abundance and tryptophan and kynurenine levels in this study suggest that post-transcriptional activity is important to this relationship. It is worth noting, however, that while the K/T ratio had reasonable test characteristics for TB in our data, it did not perform as well as other biomarkers we have assessed. Our previous analysis found that a combination of other metabolites achieved AUCs of 0.88 for diagnosis of tuberculosis and 0.86 for response to treatment. A single metabolite, N-acetylneuraminate, was found to have an AUC of 0.66 for TB diagnosis, and another, pyridoxate, was found to have an AUC of 0.87 for response to treatment (4). Similarly, a transcriptional profile derived from this dataset achieved higher sensitivity when applied to other published datasets for both diagnosis (up to 85% sensitive) and treatment response (up to 86% sensitive) (3). Likewise, other groups have published transcriptional signatures of pediatric TB with higher AUCs than that achieved by the K/T ratio in these data for diagnosis and treatment response (AUCs of 0.76 for diagnosis and 0.77 for treatment response) (19, 20). Those analyses did not identify blood tryptophan abundance or IDO-1 expression as significant.

In any study of host-derived genetic or metabolomic biomarkers, it is possible that clinical or genetic differences between study populations could impact study results. This analysis only included data from Indian children without HIV, which may limit generalizability to other populations, including those with HIV. Similarly, this analysis did not analyze samples from people with other respiratory infections and will need to be tested in that setting before it could be used for the clinical test of TB in children. Previous studies have found the K/T ratio to be useful among people both with and without HIV, independent of HIV treatment initiation (11, 21), as well as among pregnant woman (22), people with diabetes (23), and among diverse populations in East Asia, Sub-Saharan Africa, and Eastern Europe (6, 7, 10, 11). Metabolomic testing is also currently limited by the need for highly specialized, resource-intensive sensitive testing, but like many other tools, could potentially be translated to simpler methods for near-care or point-of-care testing in the future.

Our findings suggest that plasma kynurenine and the K/T ratio may be useful biomarkers for pediatric TB. Additional studies are needed to validate these biomarkers in diverse pediatric populations and in settings with lower TB incidence. Ongoing studies in geographically diverse populations will help determine the optimal use of these biomarkers worldwide.

## Data Availability Statement

The data sets presented in this study can be found in online repositories and in the supplemental files of (4). The names of the repository/repositories and accession number(s) can be found below: https://www.ncbi.nlm.nih.gov/, PRJNA588242.

## Ethics Statement

This study was approved by the institutional review boards of Byramjee Jeejeebhoy Government Medical College, the National Institute of Research in Tuberculosis, and the Johns Hopkins University School of Medicine. Written informed consent for participation was provided by the legal guardians of all pediatric participants under the age of consent, and written informed assent was provided by all pediatric participants ≥ 8 years of age.

## Author Contributions

JT contributed to the study design, sample processing, data analysis, and manuscript preparation. MP contributed to the study design, data collection, data analysis, and manuscript preparation. HZ contributed to manuscript preparation. VK and NP contributed to study design, sample processing, data analysis, and manuscript preparation. AaK and AnK contributed to data collection and manuscript preparation. NG contributed to data analysis and manuscript preparation. VM supervised study activities and contributed to study design, data collection, and manuscript preparation. AG and PK supervised study design, data collection, analysis, and manuscript preparation. All authors contributed to the article and approved the submitted version.

## Funding

CTRIUMPH was supported by the NIH/DBT RePORT India Consortium with funding in whole or in part from the Government of India’s (GOI) Department of Biotechnology (DBT), the Indian Council of Medical Research (ICMR), the United States National Institutes of Health (NIH), National Institute of Allergy and Infectious Diseases (NIAID), Office of AIDS Research (OAR), and distributed in part by CRDF Global. This publication was made possible by support from the Johns Hopkins University Center for AIDS Research (NIH P30AI094189). AG, NG, and VM were also supported by NIH/NIAID under award number UM1AI069465. PK was supported by NIH/NIAID (K24AI143447) and the U.S. Civilian Research & Development Foundation distributed by CRDF Global. JT was supported by NIH/NIAID (K23AI135102 and R21AI122922), the NIH/Fogarty Global Health Fellows Program Consortium (R25TW009340), and the Johns Hopkins University School of Medicine Clinician Scientist Career Development Award.

## Author Disclaimer

The contents of this publication are solely the responsibility of the authors and do not represent the official views of DBT, ICMR, NIH, or CRDF Global. Any mention of trade names, commercial projects, or organizations does not imply endorsement by any of the sponsoring organizations. The funding sources had no role in the study design, data collection, data analysis, data interpretation or writing of the report.

## Conflict of Interest

The authors declare that the research was conducted in the absence of any commercial or financial relationships that could be construed as a potential conflict of interest.

## Publisher’s Note

All claims expressed in this article are solely those of the authors and do not necessarily represent those of their affiliated organizations, or those of the publisher, the editors and the reviewers. Any product that may be evaluated in this article, or claim that may be made by its manufacturer, is not guaranteed or endorsed by the publisher.
